# The effect of tacrolimus conversion from immediate- to extended-release formulation on renal function in renal transplant patients: a meta-analysis

**DOI:** 10.3389/fphar.2023.1226647

**Published:** 2023-10-04

**Authors:** Sheng Chao, Lei Jia, Kejing Zhu, Luobei Chen, Yulin Niu

**Affiliations:** Department of Organ Transplantation, The Affiliated Hospital of Guizhou Medical University, Guiyang, China

**Keywords:** renal transplant, extended-release tacrolimus, immediate-release tacrolimus, serum creatinine, estimated glomerular filtration rate

## Abstract

**Objective:** Tacrolimus formulation affects the outcomes of a renal transplant, while the effect of its immediate- to extended-release conversion remains controversial. This meta-analysis aimed to compare the renal function before and after tacrolimus immediate- to extended-release conversion in renal transplant patients.

**Methods:** PubMed, Cochrane, Embase, CNKI, CQVIP, and Wanfang databases were searched for articles regarding the effect of tacrolimus conversion from immediate- to extended-release formulation on renal function in renal transplant patients. The data on serum creatinine (Scr) or the estimated glomerular filtration rate (eGFR) before and after conversion were extracted and analyzed.

**Results:** Ten studies with 743 renal transplant patients were included. Scr was reduced after conversion versus before conversion [mean difference (MD) (95% confidence interval (CI)): -8.00 (−14.33; −1.66) μmol/L, *p* = 0.01]. However, eGFR only showed an increased trend after conversion versus before conversion (MD (95% CI): 2.21 (−1.62, 6.03) mL/min/1.73 m^2^, *p* = 0.26) but without statistical significance. Furthermore, in patients with a follow-up duration ≥48 weeks, Scr was decreased after conversion versus before conversion (*p* = 0.005), but eGFR remained unchanged (*p* = 0.68). However, in patients with a follow-up duration <48 weeks, both Scr (*p* = 0.36) and eGFR (*p* = 0.24) were not different before conversion versus after conversion. Moreover, publication bias risk was low, and robustness assessed by sensitivity analysis was generally good.

**Conclusion:** This meta-analysis favors studies indicating that the conversion of tacrolimus from an immediate-release to an extended-release formulation could improve the kidney function to some extent in renal transplant patients, and this advancement may be related to the administration period.

## 1 Introduction

Renal transplant is the optimal form of renal replacement therapy, which improves the survival and quality of life in kidney failure patients (Goldfarb, 2021). According to the Organ Procurement and Transplantation Network (OPTN) and Scientific Registry of Transplant Recipients (SRTR) 2019 Annual Data Report, the 5-year graft survival ranges from 64.6% to 90.7% in renal transplant patients (Hart et al., 2021a). Notably, antibody-mediated rejection is a crucial factor for graft loss, and renal transplant patients require lifelong immunosuppressive regimens to prevent the occurrence of rejection (Hart et al., 2021b; Lai et al., 2021; Novotny et al., 2021; Wojciechowski and Wiseman, 2021). Tacrolimus is the cornerstone immunosuppressant for renal transplant patients (Hart et al., 2021a); however, conventional tacrolimus with immediate-release formulation has some non-negligible disadvantages, such as twice-a-day intake leads to poor medication adherence in some renal transplant patients (Denhaerynck et al., 2005; Morales et al., 2012; Scheel et al., 2017); meanwhile, its narrow therapeutic window and higher inter- and intra-patient pharmacokinetic variability result in a higher risk of drug-related adverse events and allograft rejection (Banas et al., 2020; Noble et al., 2021; Meera et al., 2023); these factors may further contribute to the poor outcomes of grafts and reduced patients’ quality of life after renal transplant (Banas et al., 2020). Therefore, optimizing the tacrolimus application strategy to maintain the renal function is critical for renal transplant patients.

There are two once-daily formulations of tacrolimus, namely, standard prolonged-release tacrolimus (such as Advagraf^®^) and extended-release tacrolimus (such as Envarsus^®^), which enhance the convenience for renal transplant patients and assist in improving the medical adherence of these patients (McCormack, 2014; Garnock-Jones, 2015; Tremblay and Alloway, 2017; Faravardeh et al., 2021; Budde et al., 2022). The difference between these two formulations is that extended-release tacrolimus uses MeltDose^®^ Technology, which improves the solubility of tacrolimus by dispersing it in a polymer matrix, thereby increasing its bioavailability (McCormack, 2014; Stifft et al., 2014; Garnock-Jones, 2015; Oberbauer et al., 2020). In recent decades, several studies have explored the benefit of tacrolimus conversion in improving the renal function in renal transplant patients; however, inconsistency in results exists among these studies (Gaber et al., 2013; Abedini et al., 2018; Rubik et al., 2019; Wenping et al., 2019; Jing et al., 2020; Chenguang et al., 2021; Hugo et al., 2021; Xiaohong et al., 2021; Haiwei et al., 2022; Ziyu et al., 2022). For instance, one study indicates that serum creatinine (Scr) is reduced and the estimated glomerular filtration rate (eGFR) is increased after tacrolimus conversion from immediate- to extended-release formulation in renal transplant patients (Xiaohong et al., 2021). However, two studies explain that Scr and eGFR are unchanged before and after tacrolimus conversion in renal transplant patients (Abedini et al., 2018; Jing et al., 2020).

Accordingly, although the number of relevant studies that reported the effect of tacrolimus conversion from immediate- to extended-release formulation on renal function after renal transplant was not high, this meta-analysis still aimed to provide an objective view of the impact of tacrolimus conversion on enhancing the renal function in renal transplant patients.

## 2 Methods

### 2.1 Study searching

The Preferred Reporting Item for Systematic Reviews and Meta-Analyses (PRISMA) protocol was used for this meta-analysis (Hutton et al., 2015). Studies that examined the effect of tacrolimus conversion from an immediate-release formulation to an extended-release formulation on the renal function in renal transplant patients were searched and included. The databases of PubMed, Cochrane, Embase, CNKI, CQVIP, and Wanfang were adopted. The keywords of ‘tacrolimus,’ ‘Prograf,’ ‘Prograft,’ ‘FR900506,’ ‘FK506,’ ‘extended,’ ‘prolonged,’ ‘sustained,’ ‘delayed,’ ‘convert,’ ‘conversion,’ ‘switch,’ ‘renal,’ and ‘kidney’ were used.

### 2.2 Eligibility criteria

Studies were considered eligible if they met the following criteria: 1) they enrolled renal transplant patients; 2) they evaluated the effect of tacrolimus conversion from an immediate-release formulation to an extended-release formulation on renal function; and 3) they reported the data on Scr or eGFR before and after conversion. The exclusion criteria were as follows: 1) they compared the effect of immediate-release and extended-release formulations on renal function in two separate groups, and the treatment was not converted between immediate-release and extended-release; 2) the data were unextractable and unavailable for this meta-analysis; and 3) case reports, reviews, meta-analyses, or animal studies.

### 2.3 Data extraction

Study searching, data review, and the risk of bias evaluation were conducted by two investigators. Disagreements were resolved by consensus. To be specific, titles and abstracts of studies that were thought to be relevant to the current study were evaluated by the researchers, and after that, appropriate studies were found through full-text evaluation based on the inclusion and exclusion criteria. Additionally, references for the included studies were checked. After identifying the study, the information was extracted, including the author name, year, sample size, age, gender, country, follow-up duration, tacrolimus dose, blood through level, and outcomes (Scr and eGFR). The methodological quality of the included studies was evaluated per the methodological index for non-randomized studies (MINORS), which consisted of eight items and scored 0–16 (Slim et al., 2003).

### 2.4 Statistical analysis

The Review Manager (RevMan) v5.0 software application (the Cochrane Collaboration, Denmark) was utilized. Mean differences (MDs) with 95% confidence intervals (CIs) were used for evaluation. Fixed-effect models were chosen when heterogeneity was minimal (I^2^ ≤ 50.0% and/or *p* ≥ 0.05), but random-effect models were chosen when heterogeneity was moderate or high (Higgins et al., 2003). The “leave-one-out” method was adopted for sensitivity analysis, which is conducted by sequentially omitting each study and repeating the analysis. The publication bias was investigated using fennel plots, Begg’s test, and Egger’s test. A *p* < 0.05 was considered statistically significant.

## 3 Results

### 3.1 Study screening process

**FIGURE 1:**
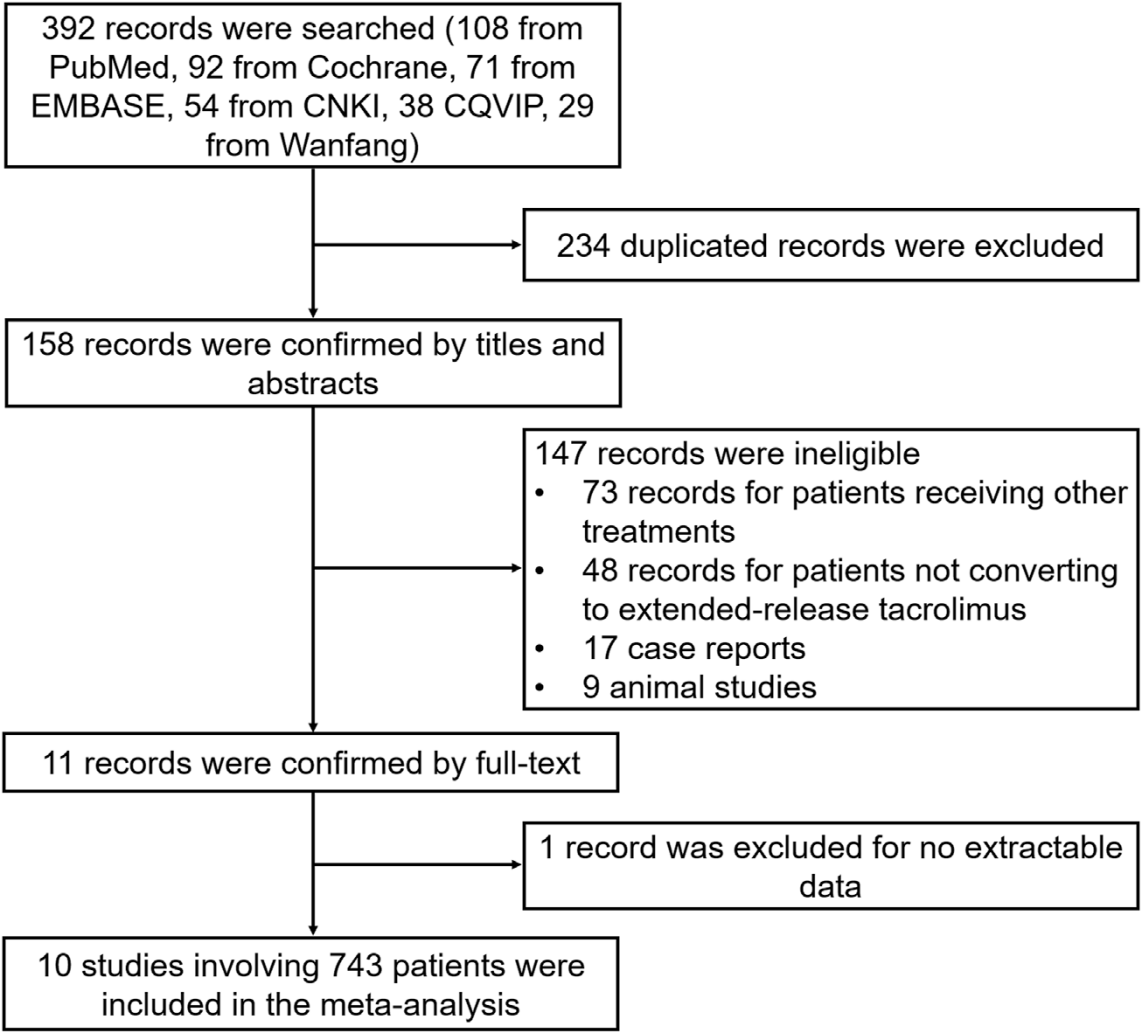
Study flow.

A total of 392 records were searched (108 records from PubMed, 92 records from Cochrane, 71 records from Embase, 54 records from CNKI, 38 records from CQVIP, and 29 records from Wanfang), and then, 234 duplicated records were excluded. Subsequently, 158 records were screened by titles and abstracts, and 147 records met the exclusion criteria, comprising 73 records for patients receiving other treatments, 48 records for patients not converting to extended-release tacrolimus, 17 case reports, and 9 animal studies. Afterward, 11 records were screened by full-text, and one record was further excluded for no extractable data. Ultimately, 10 records with 743 patients were included in this meta-analysis (Figure 1).

### 3.2 Features of screened studies

The included 10 studies were published from 2013 to 2022, which contained 743 renal transplant patients (Gaber et al., 2013; Abedini et al., 2018; Rubik et al., 2019; Wenping et al., 2019; Jing et al., 2020; Chenguang et al., 2021; Hugo et al., 2021; Xiaohong et al., 2021; Haiwei et al., 2022; Ziyu et al., 2022). Regarding countries, six studies were conducted in China, and the remaining four studies were conducted in America, Norway, Poland, and Germany, respectively. The follow-up durations ranged from 3 to 144 weeks. The detailed characteristics of the included studies are exhibited in Table 1.

**TABLE 1:** Details of the included studies.

Author	Year	Sample size	Age, mean ± SD	Male, No. (%)	Country	Follow-up duration	Tacrolimus dose (mg/d)		Blood trough level (ng/mL)		Outcome
							Before conversion	After conversion	Before conversion	After conversion	
Gaber et al. (2013)	2013	47	45.6 ± NA	32 (68.1)	America	3 weeks	NA	NA	NA	NA	eGFR
Abedini et al. (2018)	2018	91	47.7 ± 14.3	58 (63.7)	Norway	48 weeks	4.4 ± 2.5	4.4 ± 2.4	6.0 ± 1.4	5.5 ± 1.8	eGFR
Rubik et al. (2019)	2019	48	11.0 ± 3.0	29 (60.4)	Poland	54 weeks	4.5 ± 2.3	4.1 ± 2.0	5.6 ± 1.7	5.6 ± 1.5	eGFR
Wenping et al. (2019)	2019	68	37.0 ± 11.0	41 (60.3)	China	24 weeks	NA	NA	NA	NA	Scr, eGFR
Jing et al. (2020)	2020	61	47.5 ± 10.3	24 (39.3)	China	4 weeks	3.4 ± 1.3	4.0 ± 1.4	NA	NA	Scr
Hugo et al. (2021)	2021	183	51.2 ± 12.7	112 (61.2)	Germany	48 weeks	NA	NA	6.8 ± 2.0	5.6 ± 1.3	eGFR
Xiaohong et al. (2021)	2021	83	43.2 ± 10.9	57 (68.7)	China	144 weeks	2.1 ± 0.8	2.1 ± 0.8	7.2 ± 2.8	6.1 ± 2.1	Scr and eGFR
Chenguang et al. (2021)	2021	22	10.6 ± NA	14 (63.6)	China	48 weeks	3.8 ± 2.3	4.0 ± 2.4	6.2 ± 0.9	7.3 ± 1.7	Scr
Ziyu et al. (2022)	2022	39	NA	NA	China	48 weeks	NA	NA	7.1 ± 3.1	6.9 ± 1.7	Scr
Haiwei et al. (2022)	2022	101	41.9 ± 10.4	62 (61.4)	China	20 weeks	3.3 ± 1.3	4.5 ± 1.9	6.4 ± 2.3	6.8 ± 1.7	Scr

SD, standard deviation; NA, not accessible.

### 3.3 Scr and eGFR

**FIGURE 2:**
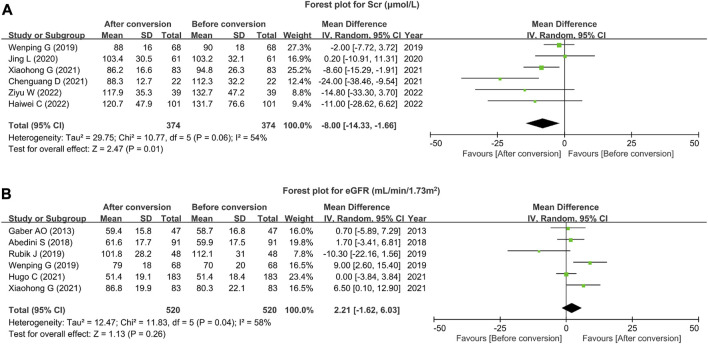
Scr and eGFR after tacrolimus conversion from immediate- to extended-release formulation in renal transplant patients. Forest plot for Scr **(A)** and eGFR **(B)** after tacrolimus conversion from immediate- to extended-release formulation in renal transplant patients.

A total of six studies reported Scr, and heterogeneity existed among these studies (I^2^ = 54%, *p* = 0.06). The pooled analysis disclosed that Scr was reduced after conversion versus before conversion in renal transplant patients (MD (95% CI): −8.00 (−14.33, −1.66) μmol/L, *p* = 0.01) (Figure 2A). In addition, there were six studies that reported eGFR, and heterogeneity existed among these studies (I^2^ = 58%, *p* = 0.04). The pooled analysis suggested that eGFR only showed a trend to increase after conversion versus before conversion in renal transplant patients (MD (95% CI): 2.21 (−1.62, 6.03) mL/min/1.73 m^2^, *p* = 0.26) (Figure 2B) but without statistical significance.

### 3.4 Subgroup analysis for Scr and eGFR

**FIGURE 3:**
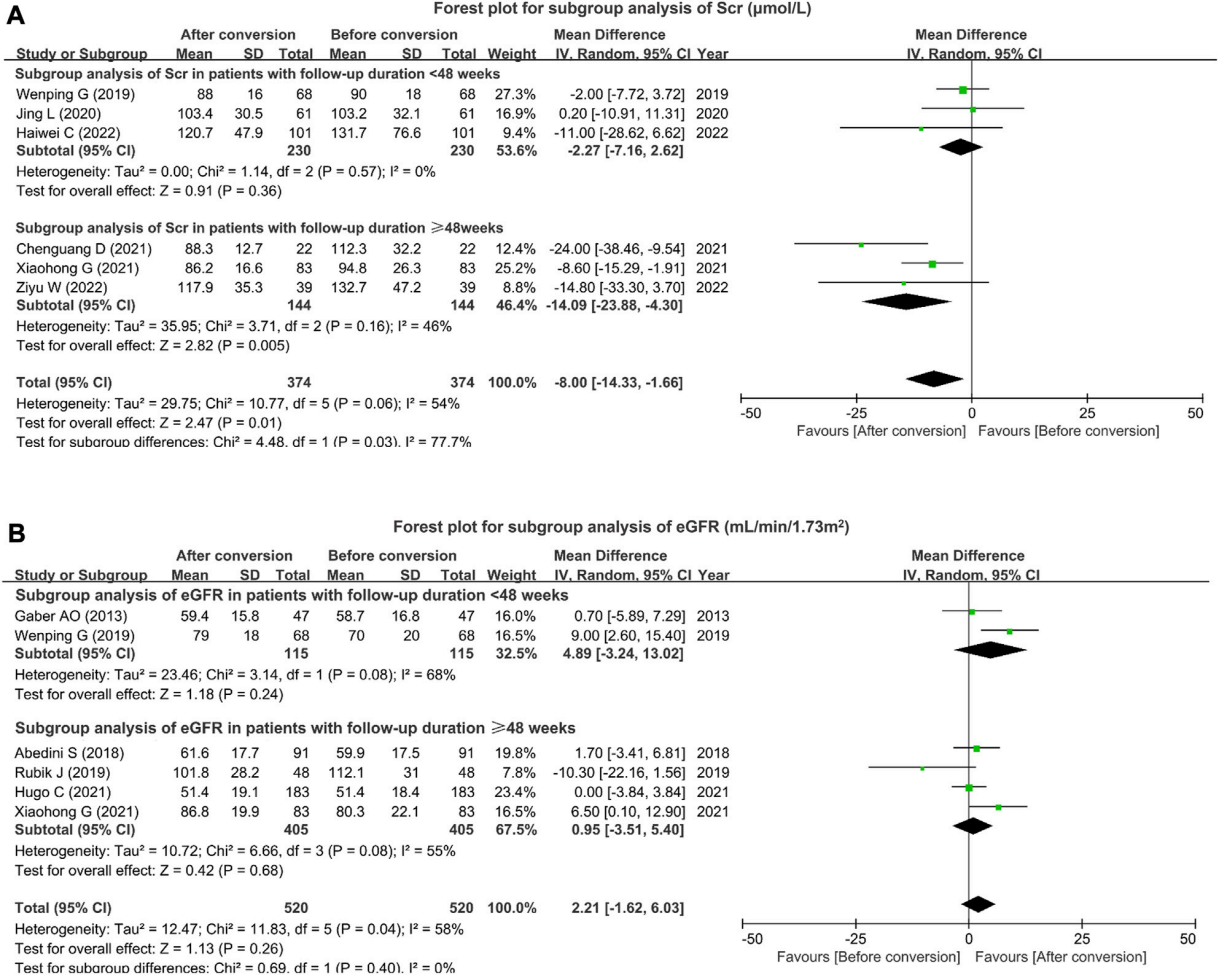
Subgroup analysis of Scr and eGFR based on the follow-up duration. Forest plot for subgroup analysis of Scr **(A)** and eGFR **(B)** based on the follow-up duration after tacrolimus conversion from immediate- to extended-release formulation in renal transplant patients.

Subgroup analysis on Scr was conducted based on the follow-up duration. Regarding studies with follow-up duration <48 weeks, a total of three studies reported Scr. Heterogeneity did not exist among these studies (I^2^ = 0%, *p* = 0.57). The pooled analysis suggested that Scr remained unchanged before and after tacrolimus formulation conversion in renal transplant patients (MD (95% CI): −2.27 (−7.16, 2.62) μmol/L, *p* = 0.36). With respect to studies with follow-up duration ≥48 weeks, the other three studies reported Scr. Heterogeneity did not exist among these studies (I^2^ = 46%, *p* = 0.16). The pooled analysis displayed that Scr was decreased after conversion versus before conversion in renal transplant patients (MD (95% CI): −14.09 (−23.88, −4.30) μmol/L, *p* = 0.005) (Figure 3A).

Subgroup analysis on eGFR was also conducted based on the follow-up duration. In terms of studies with follow-up duration <48 weeks, two studies reported eGFR. Heterogeneity existed among these studies (I^2^ = 68%, *p* = 0.08). eGFR was not different before and after tacrolimus formulation conversion in renal transplant patients (MD (95% CI): 4.89 (−3.24, 13.02) mL/min/1.73 m^2^, *p* = 0.24). Regarding studies with follow-up duration ≥48 weeks, four studies reported eGFR. Heterogeneity existed among these studies (I^2^ = 55%, *p* = 0.08). The pooled analysis suggested that no difference in eGFR was found before and after tacrolimus formulation conversion in renal transplant patients (MD (95% CI): 0.95 (−3.51, 5.40) mL/min/1.73 m^2^, *p* = 0.68) (Figure 3B).

Subgroup analysis on Scr and eGFR was conducted based on the tacrolimus dose after conversion. In patients with the tacrolimus dose after conversion ≤4.0 mg/d, three studies reported Scr. Heterogeneity existed among these studies (I^2^ = 70%, *p* = 0.03). The pooled analysis disclosed that Scr showed a decreasing trend after tacrolimus conversion versus before conversion but did not achieve statistical significance (MD (95% CI): −9.82 (−20.94, 1.30) μmol/L, *p* = 0.08). Unfortunately, in patients with the tacrolimus dose after conversion >4.0 mg/d, only one study reported Scr, and Scr was not changed before and after tacrolimus conversion (MD (95% CI): −11.00 (−28.62, 6.62) μmol/L, *p* = 0.22) (Supplementary Figure S1A). In patients with the tacrolimus dose after conversion ≤4.0 mg/d, only one study reported eGFR, and eGFR exhibited an increasing trend after tacrolimus conversion compared to before conversion but did not reach statistical significance (MD (95% CI): 6.50 (0.10, 12.90) mL/min/1.73 m^2^, *p* = 0.05). In patients with the tacrolimus dose after conversion >4.0 mg/d, two studies reported eGFR. Heterogeneity existed among these studies (I^2^ = 70%, *p* = 0.07). The pooled analysis suggested that eGFR was not different before and after tacrolimus conversion (MD (95% CI): −3.06 (−14.56, 8.45) mL/min/1.73 m^2^, *p* = 0.60) (Supplementary Figure S1B).

Subgroup analysis on Scr and eGFR was also performed based on the tacrolimus blood trough level after conversion. In patients with the tacrolimus blood trough level after conversion ≤6.0 ng/mL, no studies reported Scr. In patients with the tacrolimus blood trough level after conversion >6.0 ng/mL, four studies reported Scr. Heterogeneity did not exist among these studies (I^2^ = 19%, *p* = 0.29). The pooled analysis disclosed that Scr was decreased after conversion (MD (95% CI): −12.62 (−19.53, −5.71) mL/min/1.73 m^2^, *p* = 0.0003) (Supplementary Figure S2A). Additionally, in patients with the tacrolimus blood trough level after conversion ≤6.0 ng/mL, three studies reported eGFR. Heterogeneity did not exist among these studies (I^2^ = 40%, *p* = 0.19). The pooled analysis suggested that eGFR was not affected by tacrolimus conversion (MD (95% CI): -0.52 (−4.84, 3.79) mL/min/1.73 m^2^, *p* = 0.81). In patients with the tacrolimus blood trough level after conversion >6.0 ng/mL, only one study reported eGFR, and eGFR showed an increasing trend after tacrolimus conversion versus before conversion but did not achieve statistical significance (MD (95% CI): 6.50 (0.10, 12.90) mL/min/1.73 m^2^, *p* = 0.05) (Supplementary Figure S2B).

### 3.5 Quality assessment

**FIGURE 4:**
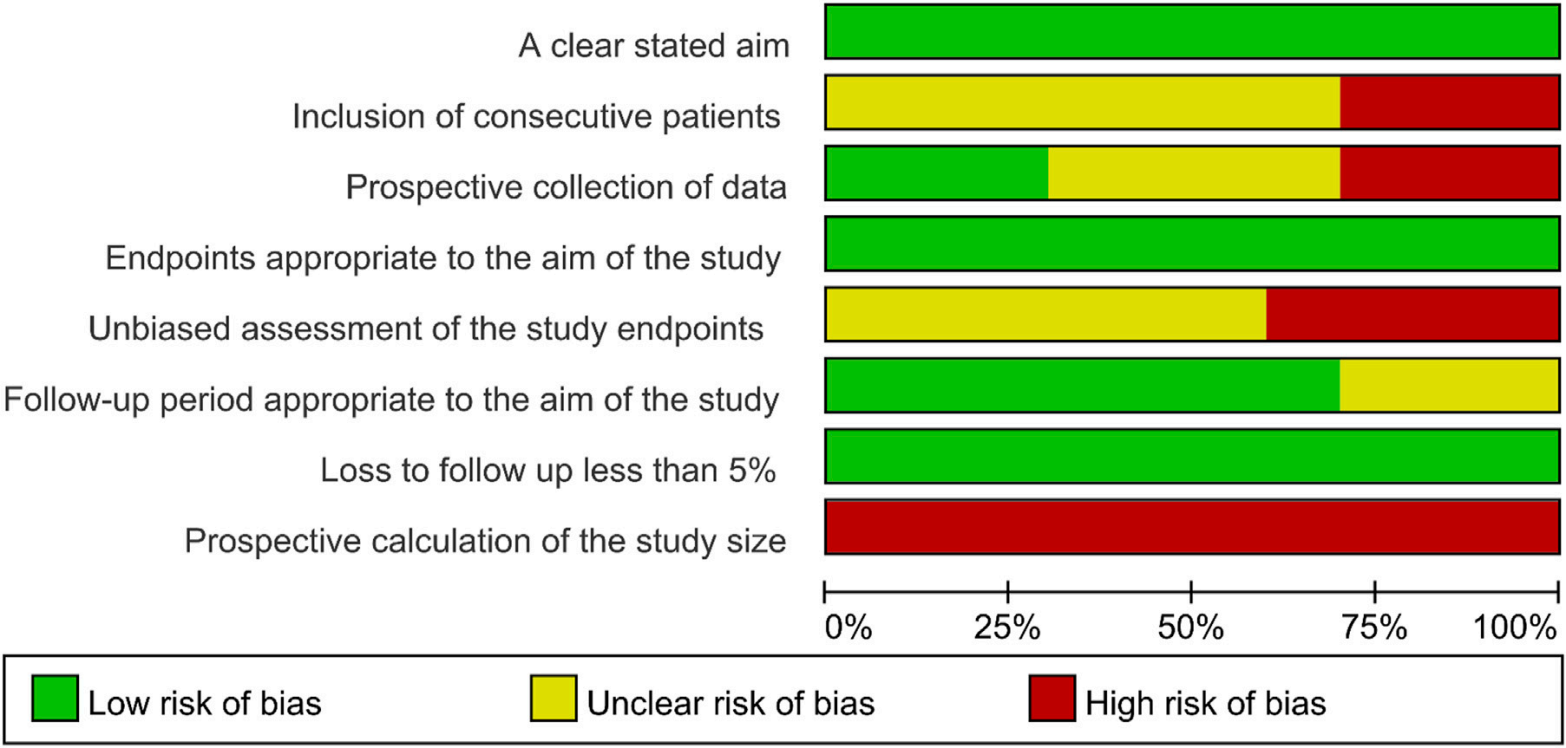
Risk of bias assessment.

All included studies had a low risk of bias regarding a clearly stated aim, endpoints appropriate to the aim of the study, and loss to follow-up of less than 5%. In addition, a proportion of studies (less than 50%) had an unclear risk of bias regarding the follow-up period appropriate to the aim of the study. Moreover, more than 25% of studies had a high bias risk of inclusion of consecutive patients, prospective collection of data, and unbiased assessment of the study endpoints. Notably, all studies had a high risk of bias regarding the prospective calculation of the study size (Figure 4).

### 3.6 Publication bias and sensitivity analysis

**FIGURE 5:**
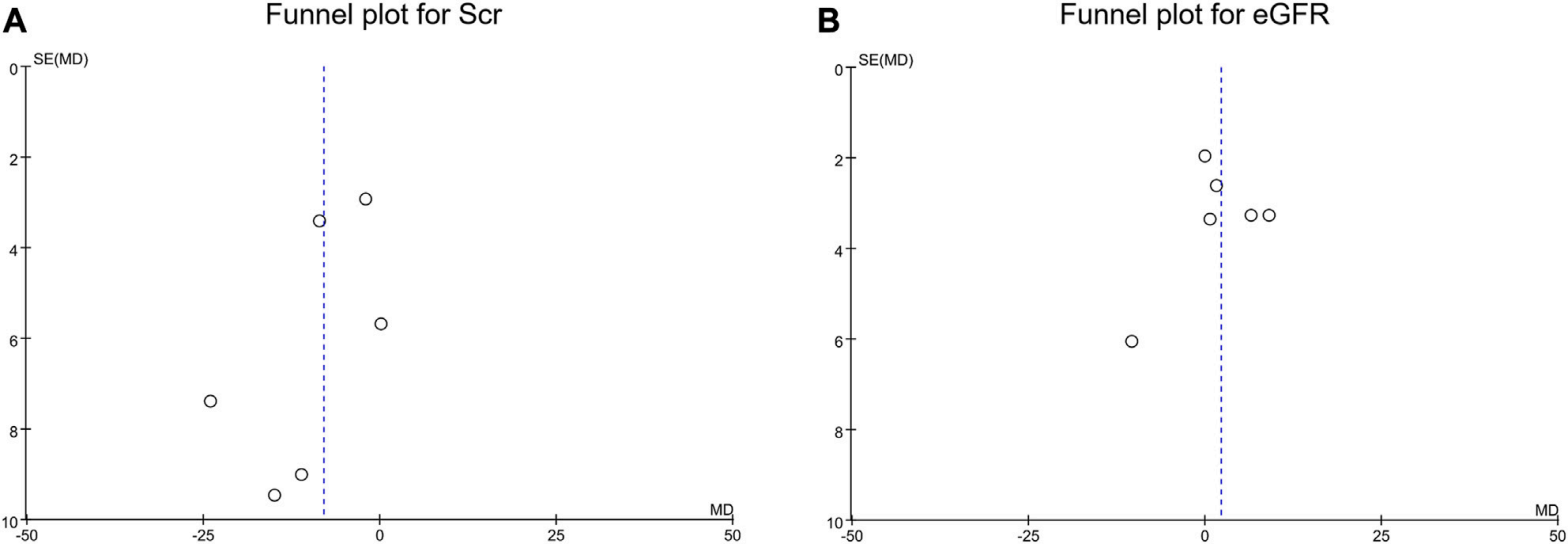
Publication bias. Funnel plot for Scr **(A)** and eGFR **(B)**.

According to the fennel plots, the publication bias of Scr (Figure 5A) and eGFR (Figure 5B) was generally low. Meanwhile, Begg’s test and Egger’s test further validated that no publication bias of Scr and eGFR existed (all *p* > 0.05).

Sensitivity analysis revealed that omitting Xiaohong G (2021) would affect the result of Scr. Apart from that, the MD of Scr and eGFR would not be affected by omitting any of the other studies, indicating the robustness of this meta-analysis (Table 2).

**TABLE 2:** Sensitivity analysis.

Omitted study	MD	95% CI	
		Lower	Upper
**Scr (μmol/L)**			
Wenping G (2019)	−10.27	−17.78	−2.76
Jing L (2020)	−9.83	−17.07	−2.60
Xiaohong G (2021)	−8.53	−17.25	0.18
Chenguang D (2021)	−5.08	−9.41	−0.75
Ziyu W (2022)	−7.42	−14.25	−0.58
Haiwei C (2022)	−7.86	−14.93	−0.79
Combined	−8.00	−14.33	−1.66
**eGFR (mL/min/1.73 m** ^**2**^ **)**			
Gaber AO (2013)	2.43	−2.18	7.04
Abedini S (2018)	2.21	−2.71	7.13
Rubik J (2019)	3.12	−0.25	6.48
Wenping G (2019)	1.00	−2.46	4.46
Hugo C (2021)	2.75	−2.08	7.57
Xiaohong G (2021)	1.35	−2.89	5.58
Combined	2.21	−1.62	6.03

MD, mean difference; CI, confidence interval; Scr, serum creatinine; eGFR, estimated glomerular filtration rate.

## 4 Discussion

Tacrolimus conversion from immediate- to extended-release formulation has certain implications on the renal function, according to previous studies (Wenping et al., 2019; Jing et al., 2020; Chenguang et al., 2021; Xiaohong et al., 2021; Haiwei et al., 2022; Ziyu et al., 2022); however, inconsistency exists among these studies. In this meta-analysis, it was found that renal function (reflected by Scr) was improved, following tacrolimus conversion from immediate- to extended-release formulation in renal transplant patients. The potential reasons would be as follows (Goldfarb, 2021): an extended-release tacrolimus formulation might have a flat pharmacokinetic profile, such as higher bioavailability, less fluctuation between trough and peak exposures, and a delayed peak concentration; thus, it might have a better effect on inhibiting antibody-mediated rejection, thus improving the renal function (Garnock-Jones, 2015; Oberbauer et al., 2020; Hart et al., 2021a). An extended-release tacrolimus formulation only needs to be taken once daily, which might increase medication adherence, and then improved the renal function (Singh et al., 2015; Oberbauer et al., 2020). Another finding of this meta-analysis should also be noticed. As an important renal function index, eGFR only showed an increasing trend after tacrolimus conversion but did not reach statistical significance. Further studies are warranted to verify the findings of this meta-analysis. Notably, it should be clarified that the types of extended-release tacrolimus are different in the included studies, which included Envarsus^®^ and Advagraf^®^ (Gaber et al., 2013; Abedini et al., 2018; Rubik et al., 2019; Hugo et al., 2021). Six included studies did not report the information on the type of extended-release tacrolimus (Wenping et al., 2019; Jing et al., 2020; Chenguang et al., 2021; Xiaohong et al., 2021; Haiwei et al., 2022; Ziyu et al., 2022).

Apart from the aforementioned findings, this meta-analysis also carried out subgroup analyses to confirm the effect of tacrolimus conversion from immediate- to extended-release formulation on improving renal function in renal transplant patients. It was found that based on the result of Scr, renal function was improved after tacrolimus conversion from immediate- to extended-release formulation in renal transplant patients with a follow-up duration ≥48 weeks but not in patients with a follow-up duration <48 weeks. The possible interpretation would be that, as discussed previously, extended-release tacrolimus was in a once-daily formulation procedure, which increased medication adherence (Hatakeyama et al., 2012; Singh et al., 2015; Oh et al., 2020; Verma et al., 2023); therefore, the long-term effect of conversion to extended-release tacrolimus on improving the renal function in renal transplant patients was obvious. Further subgroup analysis discovered that in patients with the tacrolimus dose after conversion ≤4.0 mg/d and patients with the tacrolimus blood trough level after conversion >6.0 ng/mL, the renal function seemed to be improved. The potential reasons could be that (Goldfarb, 2021) tacrolimus had a narrow therapeutic window; the therapeutic and toxic doses were close; after conversion to the extended-release tacrolimus, high doses might have led to excessive immunosuppression, which impaired the renal function (Piotti et al., 2017; Oberbauer et al., 2020). Therefore, the tacrolimus dose after conversion ≤4.0 mg/d enhanced the renal function (Hart et al., 2021a). The tacrolimus blood trough level reflected the lowest value of steady-state blood concentrations, and trough levels below this would weaken the effects of tacrolimus, thus impairing the renal function (Piotti et al., 2017). Hence, renal function was improved in patients with the tacrolimus blood trough level after conversion >6.0 ng/mL. However, restricted by the number of included studies, studies that could be included in the subgroup analyses were much smaller. Thus, the findings of our subgroup analyses should be further validated.

According to the quality assessment, most risks of bias were generally low, apart from the risk of bias regarding the prospective calculation of the study size, and the explanation was hypothesized by the screened studies was single-arm; therefore, there was no need to calculate the sample size. Conclusively, the screened studies were generally of good quality for this meta-analysis. Moreover, the funnel plots for Scr and eGFR suggested that the publication bias was unobvious, which was further confirmed by Begg’s test and Egger’s test. This finding indicated the results of this meta-analysis were less likely to be under- or over-estimated.

Several limitations should be noticed in this meta-analysis. First, most of the enrolled studies had a small sample size of less than 100; thus, more large-scale studies were required to validate the findings of this meta-analysis. Second, although most risks of bias were low, more than 25% of studies had a high risk of bias regarding the inclusion of consecutive patients, prospective collection of data, and unbiased assessment of the study endpoints, which would potentially influence the results of this meta-analysis. Third, the overall follow-up duration was not long enough in the included studies; thus, the long-term effect of tacrolimus conversion on improving the renal function after renal transplant still needed further exploration. Fourth, the included studies were conducted in different countries; in detail, six studies were conducted in China, and the remaining studies were conducted in America, Norway, Poland, and Germany, respectively. Thus, possible genetic and environmental factors might affect the treatment response. Fifth, the number of included studies was relatively low in this meta-analysis, contributing a much smaller sample size of the subgroup analyses, which might reduce the robustness of this meta-analysis. Sixth, in studies with a follow-up duration of <48 weeks, only Wenping G (2019) reported both Scr and eGFR. In studies with a follow-up duration of ≥48 weeks, only Xiaohong G (2021) reported both Scr and eGFR. In the remaining the studies, only one parameter was available. Therefore, the findings of the subgroup analysis should be further validated. Seventh, the type of extended-release tacrolimus (including Envarsus^®^ and Advagraf^®^) was not unified in the included studies, which might lead to different trough levels or peak levels, thus affecting the results of this meta-analysis.

The results of this meta-analysis favor studies indicating that the conversion of tacrolimus from an immediate-release to an extended-release formulation could improve kidney function to some extent in renal transplant patients, and this effect can be related to the period of administration. Clinically, renal transplant patients could be encouraged to switch tacrolimus to the extended-release formulation, especially considering the long-term administration period of tacrolimus.
