# Supplementary figures and images for: The effect of tacrolimus conversion from immediate- to extended-release formulation on renal function in renal transplant patients: a meta-analysis
Source: Front Pharmacol. 2023 Oct 4;14:1226647. doi: 10.3389/fphar.2023.1226647 (PMC10582328; doi:10.3389/fphar.2023.1226647)

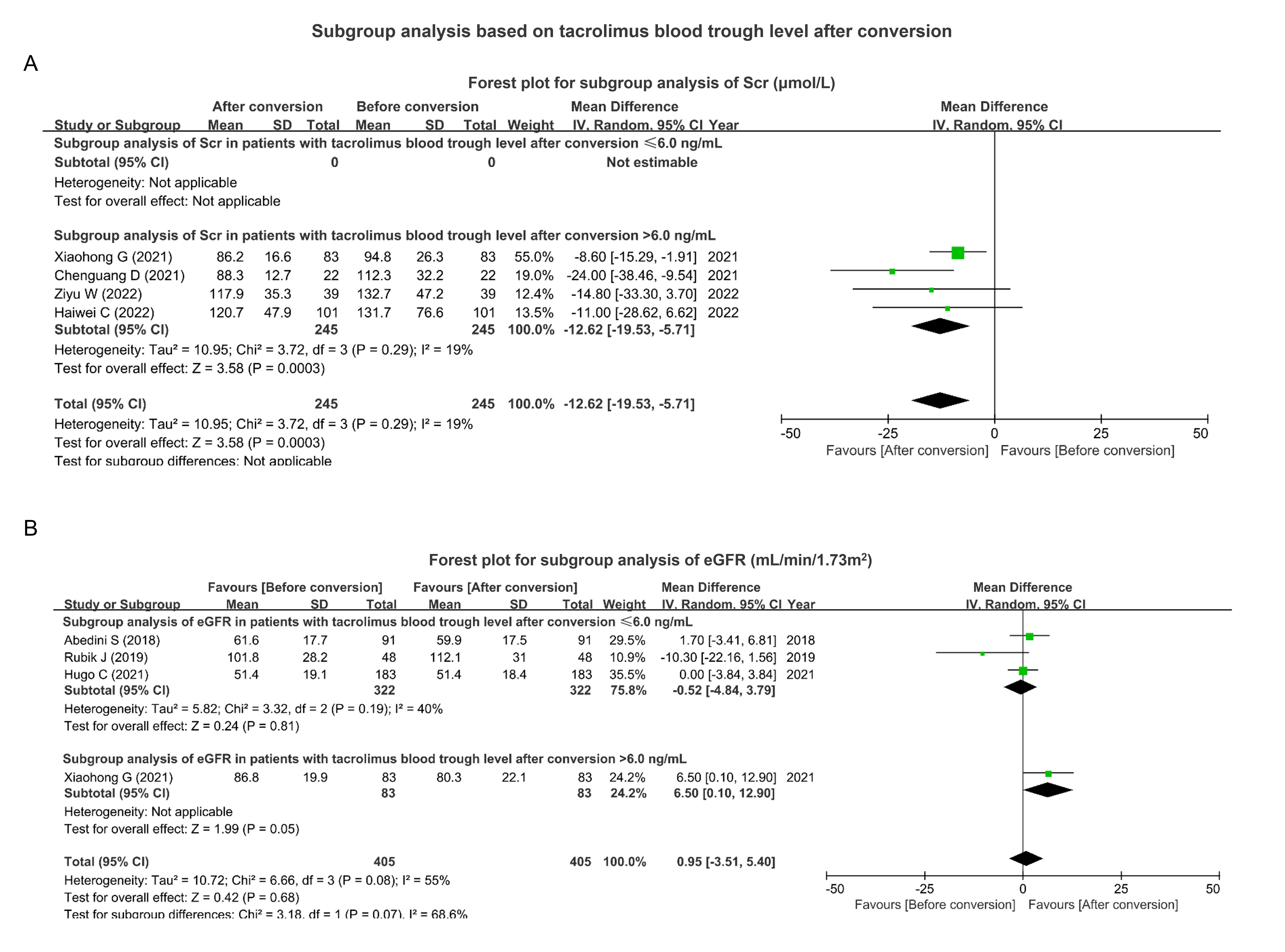

Supplement: Supplementary file 1 [file Image2.TIF]

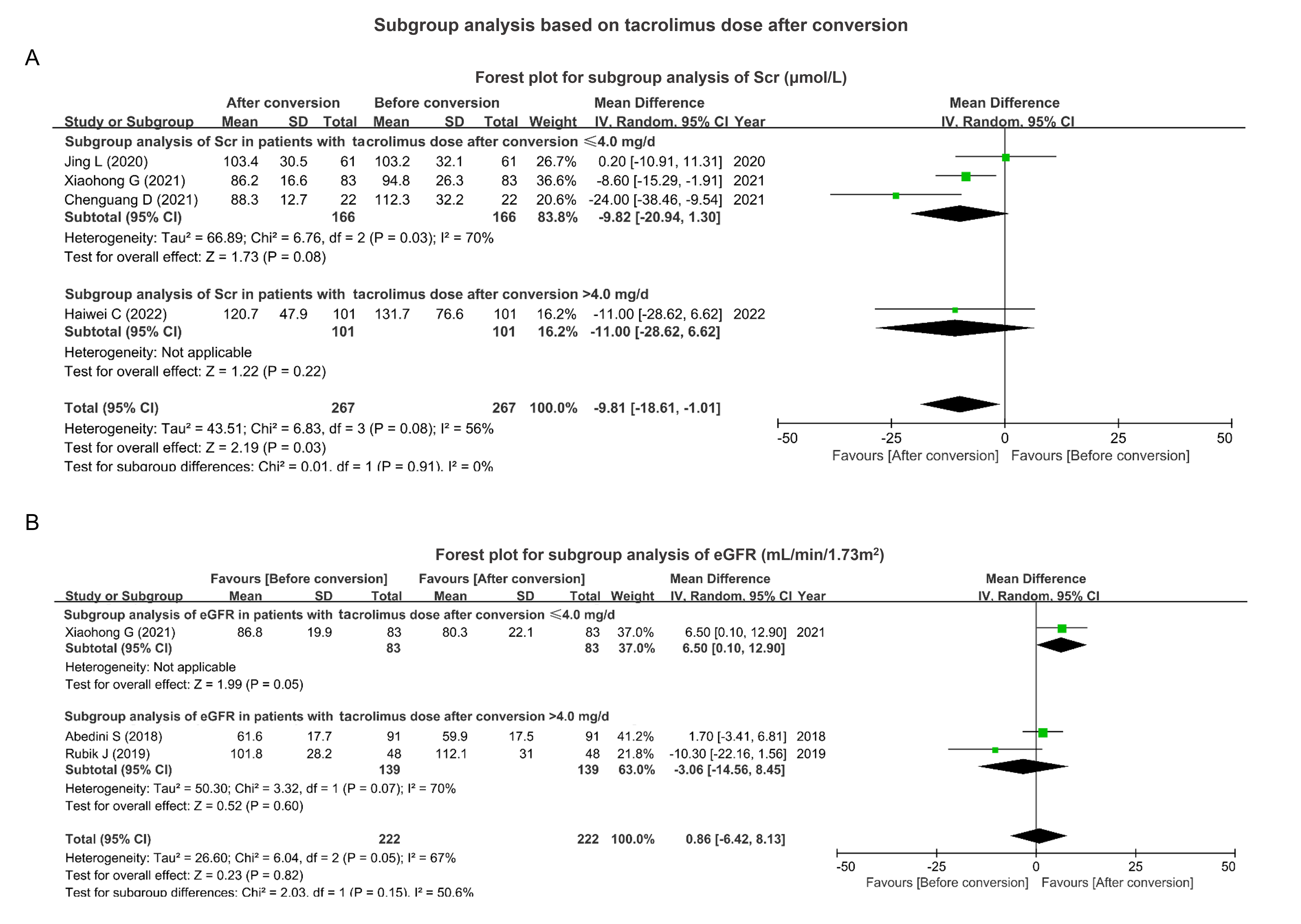

Supplement: Supplementary file 2 [file Image1.TIF]
